# A SiC Planar MOSFET with an Embedded MOS-Channel Diode to Improve Reverse Conduction and Switching

**DOI:** 10.3390/mi14071282

**Published:** 2023-06-22

**Authors:** Ping Li, Jingwei Guo, Shengdong Hu, Zhi Lin

**Affiliations:** 1 Chongqing Engineering Laboratory of High Performance Integrated Circuits, School of Microelectronics and Communication Engineering, Chongqing University, Chongqing 400044, China; li_ping_finn@163.com (P.L.); cqu_gjw@163.com (J.G.); husd@cqu.edu.cn (S.H.); 2 China Resources Microelectronics (Chongqing) Ltd., Chongqing 401331, China

**Keywords:** SiC MOSFET, MOS-channel diode, split-gate, bipolar degradation, reverse conduction

## Abstract

A novel split-gate SiC MOSFET with an embedded MOS-channel diode for enhanced third-quadrant and switching performances is proposed and studied using TCAD simulations in this paper. During the freewheeling period, the MOS-channel diode with a low potential barrier constrains the reverse current flow through it. Therefore, the suggested device not only has a low diode cut-in voltage but also entirely suppresses the intrinsic body diode, which will cause bipolar deterioration. In order to clarify the barrier-lowering effect of the MOS-channel diode, an analytical model is proposed. The calibrated simulation results demonstrate that the diode cut-in voltage of the proposed device is decreased from the conventional voltage of 2.7 V to 1.2 V. In addition, due to the split-gate structure, the gate-to-drain charge (*Q*_GD_) of the proposed device is 20 nC/cm^2^, and the reverse-transfer capacitance (*C*_GD_) is 14 pF/cm^2^, which are lower than the *Q*_GD_ of 230 nC/cm^2^ and the *C*_GD_ of 105 pF/cm^2^ for the conventional one. Therefore, a better high-frequency figure-of-merit and lower switching loss are obtained.

## 1. Introduction

Because of their faster switching speed, higher temperature operation, and lower switching loss, silicon carbide metal–oxide–semiconductor field-effect transistors (SiC MOSFETs) are very promising candidates to replace silicon insulated gate bipolar transistors (IGBTs) in medium and high voltage ranges [1,2]. In most power switching applications, an anti-paralleled freewheeling diode is required to conduct the reverse current [3]. Alternatively, there is a cost-saving and area-effective method for the application of the MOSFET by using the internal body PiN diode to conduct the reverse current [4,5]. However, the wide bandgap of materials means the body PiN diode of the SiC MOSFET has a high turn-on voltage, which increases the reverse conduction losses [6,7]. Worse still, the operation of the body PiN diode of the SiC MOSFETs would cause serious reliability problems due to the basal plane dislocations (BPDs) [8,9].

Due to these two factors, the SiC MOSFET body diode is inappropriate for use as a freewheeling diode. Existing strategies for enhancing the subpar characteristics of the SiC MOSFET body diode can be categorized into two kinds: The first strategy concentrates on establishing a low turn-on voltage conduction path external to the MOSFET circuitry. This is accomplished through the use of an external anti-paralleled Schottky barrier diode (SBD) or synchronous rectification [10,11]. However, the incorporation of an external SBD increases parasitic inductance, capacitance, and overall chip area [12]. Synchronous rectification, on the other hand, necessitates the presence of two gate electrodes, which raises the cost of the system. In addition, the body diode continues to conduct during the dead time, making it difficult to achieve a balance between system security and switching losses [13]. The second strategy entails enhancing the device’s internal structure to suppress the body diode’s conduction inherently, which offers significant advantages. On the one hand, integrating a diode into a SiC MOSFET makes it possible to share the same drift region and terminal region, resulting in a significant reduction in total chip area. In addition, integrating the diode within the SiC MOSFET decreases the number of components required for the module and their interconnections, thereby reducing parasitic capacitance and inductance [14]. Ultimately, this results in increased power density and decreased switching losses. Therefore, integrated devices represent the future orientation for the development of SiC MOSFETs [15].

Various integrated devices are suggested and demonstrated to improve the characteristics of the body diode in SiC MOSFETs through simulations or fabrications. Among them, integrating a Schottky diode (SBD or junction barrier Schottky diode) is widely adopted [16,17,18,19]. However, the high-temperature leakage current of the Schottky diode is much larger than that of the PiN diode. Also, the Schottky diode could affect the reliability in some extreme operating conditions, even though the Schottky contact is well-protected from a high electric field [20]. It has been demonstrated that diode-integrated MOSFETs use the same MOS-channel to conduct forward and reverse currents [21,22]. However, a thin and heavily doped N-type epitaxial layer is required, which may cause process problems and reliability issues [16]. The SiC MOSFET with a built-in MOS-channel diode has been reported, but the relatively thin gate oxide may induce the oxide reliability issue [5,23]. A SiC MOSFET with an embedded low barrier diode has also been proposed, which requires an additional N-base region with a complicated process [24,25].

It has been experimentally demonstrated that adopting the accumulation channel and shortening the channel length are capable of reducing the third-quadrant diode voltage drop due to a low channel potential barrier [26,27]. However, the parasitic body diode is still turned on at around 2.7 V during the reverse conduction state and the blocking characteristics are devastating due to the greater leakage current that would be produced [28]. In this paper, a split-gate (SG) SiC MOSFET with an embedded MOS-channel diode is proposed to suppress the bipolar conduction of the body PiN diode and to improve the switching performance. The realization of the small potential barrier for electrons for the MOS-channel diode is achieved by shortening the channel length and utilizing a source-connected dummy gate (DG). The inversion-channel and thick gate oxide thickness are preserved; the device reliability therefore has no degradation in the proposed MOSFET. The device mechanisms and electric characteristics are investigated using TCAD Sentaurus [29] and a simplified analytical model. This paper also discusses the good reliability of the proposed device against short-circuit stress.

## 2. Device Structure and Mechanism

**Figure 1:**
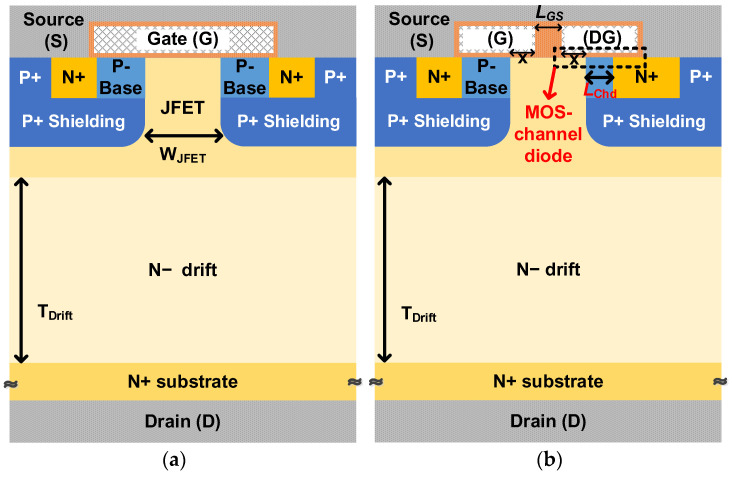
Schematic cross-section views of (**a**) conventional SiC MOSFET and (**b**) the proposed SiC MOSFET.

The schematic cross-section of the proposed SiC MOSFET (Prop. MOS) is shown in Figure 1b. Compared with the conventional SiC MOSFET (Conv. MOS), which is shown in Figure 1a, the proposed one features an embedded MOS-channel diode with an asymmetric cell structure. The MOS-channel diode is composed of an N^+^ source region, a split dummy gate (short connected to the source electrode), a P-base, and a JFET region. The channel length of the MOS-channel diode can be adjusted by only varying the length of the N^+^ source region. Obviously, the MOS-channel diode can be produced without the need for an extra mask or complicated fabrication. Additionally, the split-gate technology, which could improve the high-frequency figures-of-merit (HF-FOMs) without much reliance on special processes, has been demonstrated by many experimental validations [30,31,32]. Therefore, the proposed SiC MOSFET would not increase the difficulty of fabrication.

Both of the two devices under study are 1.2 kV-capable, and the thickness and doping concentration of the drift region are 10 μm and 8 × 10^15^ cm^−3^, respectively. To prevent punch-through during reverse bias, a heavily doped P^+^ shielding region beneath the P- base was used [26]. The doping concentration of the JFET region was selected at a relatively high value of 1 × 10^16^ cm^−3^, which could reduce the JFET on-resistance. The active channel length was 0.5 μm, and the *L*_Chd_ was optimized for the proposed SiC MOSFET. The extension of the split gate over the P-base region, which is denoted as X in Figure 1b, is a very influential dimension in the electric field at the gate corner (*E*_ox_) and gate oxide capacitance (*C*_ox_). Accordingly, the value of the X was chosen at 0.3 μm, which is an optimized value for an excellent tradeoff between the *E*_ox_ and *C*_ox_ [33]. It is noteworthy that the oxide thickness of the MOS-channel diode is the same as the gate oxide, and both of them are 50 nm. The other key device dimensions and doping values are listed in Table 1.

**Figure 2:**
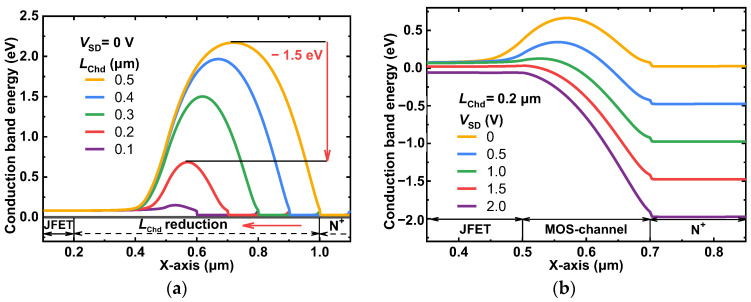
Dependence of the conduction band distribution along the SiC/oxide interface on (**a**) the length of the channel and (**b**) the positive dummy gate bias.

The positive dummy gate bias (*V*_SD_) and the channel length of the MOS-channel diode (*L*_Chd_) are two characteristics that are essential for lowering the potential barrier. The dependence of the MOS-channel diode conduction band distribution and the SiC/oxide interface on *L*_Chd_ and *V*_SD_ are shown in Figure 2a,b, respectively. When *L*_Chd_ is reduced from 0.5 μm to 0.2 μm, the electron potential barrier can be reduced by 1.5 eV at zero bias, and when *V*_SD_ is increased, the electron potential barrier could also be decreased significantly. Therefore, in the proposed SG SiC MOSFET, the intrinsic body PiN diode could be completely suppressed by a low cut-in voltage MOS-channel diode. To analyze and describe the barrier-lowering effect induced by *L*_Chd_ and *V*_SD_ of the MOS-channel diode, a compact analytical model is proposed as follows:

(1) $$V_{PB,MCD}=\frac{kT}{q}\ln\frac{N_{D}N_{A}}{n_{i}^{2}}+\frac{qN_{A}}{2_{\varepsilon \mathrm{OX}}}t_{\mathrm{eff}}t_{\mathrm{OX}}-\phi_{\mathrm{Si}\text{\_}\mathrm{SiC}}-Q_{\mathrm{OX}}\frac{t_{\mathrm{OX}}}{\varepsilon_{\mathrm{OX}}}-\frac{V_{\mathrm{SD}}}{2}$$

where *V*_PB,MCD_ denotes the potential barrier of the MOS-channel diode, *k* is Boltzmann’s constant, *T* denotes the temperature, *q* is the unit charge, *N*_D_ and *N*_A_ are the doping concentration sof the JFET and P-base region, respectively, *n*_i_ is the intrinsic doping concentration of SiC, *ε*_OX_ is the dielectric constant of the oxide, *t*_OX_ is the thickness of the oxide, *ϕ*_Si_SiC_ is the work function difference between the dummy source polysilicon and the P-base region, and *Q*_OX_ is the fixed charge at the interface of oxide and SiC. The first term on the right-hand side of Equation (1) represents the energy required for electrons to cross the JFET region. The second term indicates how much energy band bending the electric field in the P-base region has caused. The last two terms, respectively, represent the influence of fixed positive charges at the SiC/oxide interface and the effect of the source-to-drain voltage on the barrier height. It should be noted that *t*_eff_, which denotes the effective depleted thickness of the P-base region, can be expressed as:

(2) $$t_{\mathrm{eff}}=aL_{\mathrm{Chd}}^{2}+bL_{\mathrm{Chd}}+c$$

**Figure 3:**
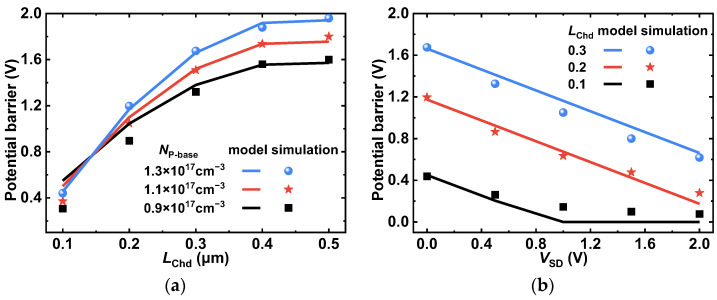
Influence of (**a**) *N*_A_ and *L*_Chd_ and (**b**) *V*_SD_ and *L*_Chd_ on the potential barrier of the MOS-channel diode.

Here, the fitting parameters of *a*, *b*, and *c* are −1.3038, 1.1997, and −0.1411, respectively. Figure 3a shows the influence of *N*_A_ and *L*_Chd_ on *V*_PB,MCD_. Figure 3b shows the influence of *V*_SD_ and *L*_Chd_ on *V*_PB,MCD_. Both of them are acquired through the proposed model and the simulations are shown. The simulation findings and the analytical model correspond well, and the levels of potential barrier reduction for *V*_SD_ and *L*_Chd_ are accurately reproduced. Obviously, as shown in Figure 3a, *V*_PB,MCD_ is reduced significantly by a quadratic function of the shortened *L*_Chd_, which can be explained by (2). Figure 3b shows that *V*_PB,MCD_ is reduced linearly with *V*_SD_ due to the enhanced MOS depletion of the surface channel [6]. However, when *V*_SD_ is larger than 1.0 V and *L*_Chd_ is equal to 0.1 μm, *V*_PB,MCD_ will not decrease significantly with *V*_SD_ because the surface region of the P-base has been completely depleted. In this case, the further reduction of *V*_PB,MCD_ that is calculated by the model is neglected. It should be noted that the proposed model is not a perfect correspondence, but it is adequate for the analysis and clarification of the mechanism of *V*_PB,MCD_ reduction for the proposed SiC MOSFET.

## 3. Results and Discussion

**Figure 4:**
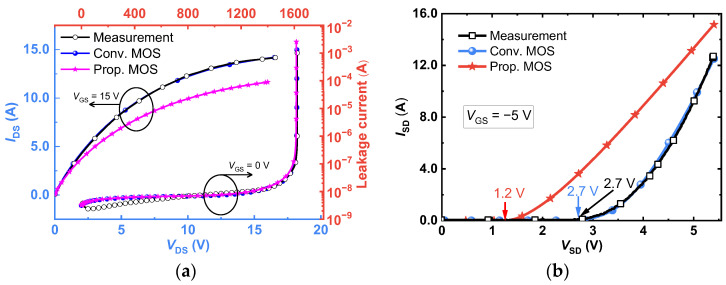
Measured and simulated characteristics of (**a**) output and breakdown and (**b**) reverse conduction.

The static and dynamic characteristics of the studied SiC MOSFETs were researched using the Sentaurus TCAD tools. In the numerical simulations, a number of crucial models that take SiC materials’ uniqueness into account were used. The Shockley–Read–Hall recombination takes the temperature and doping concentration into account. The Auger recombination, Okuto–Crowell impact ionization, incomplete dopant ionization, band narrowing, barrier lowering, and anisotropic material properties were considered. Additionally, mobility models related to doping dependence, high field saturation, and degradation at the interface were also taken into consideration. The simulation models and parameters were then calibrated to the measured *I*–*V* curves in the first and third quadrants of the commercially available Wolfspeed device (C2M0280120D [34]; see Figure 4a,b), respectively. The Conv. MOS simulation results and the measured results from the aforementioned commercial device were used to calibrate the model and its parameters. The major calibration approach for the output properties in the first quadrant entails modifying the mobility’s value and mobility model coefficients. Additionally, the thermal resistance value is changed to account for the self-heating effect brought on by an increase in the device’s output current. The impact ionization model’s coefficients were modified to fit the breakdown voltage as the primary calibration method for the breakdown characteristics. Additionally, changes were made to the carrier generation rate to meet the low leakage current. Deep-level acceptor and donor traps were placed in the P-type material region for the purpose of replicating the reverse conduction characteristics, and their energy levels were modified in accordance with pertinent literature [26].

Figure 4 also compares the *I-V* characteristics of the two SiC MOSFETs studied. When the devices work in the first quadrant, two MOSFETs have the same breakdown voltage (BV = 1615 V, *V*_GS_ = 0 V), as revealed in Figure 4a. The simulation results demonstrate that the P^+^ shielding layer provides a shielding effect against the high electric field in the channel region when the device is in the blocking state. This shielding effect helps to prevent the premature punch-through breakdown of the MOS channel diode due to its extremely low potential barrier. Based on the first-quadrant output characteristics of the *I*-*V* curve, the on-state resistance of the proposed device is higher than that of the Conv. MOS. This is because the MOS channel diode is inactive in the first-quadrant output condition, resulting in a decrease in channel density. As a consequence, the former’s *R*_on_ is 665 mΩ, while the latter’s is 510 mΩ at *V*_GS_ = 15 V and *I*_DS_ = 6 A, and the correspondingly specific on-resistance values are 13.3 mΩ·cm^2^ and 10.2 mΩ·cm^2^, respectively. Nonetheless, when compared to the Conv. MOS, the Prop. MOS’s HF-FOM greatly decreases due to the split-gate structure’s large reduction of *Q*_GD_ (*C*_GD_). In addition, even though the construction of an embedded MOS channel diode reduces the conductive channel density, it also lowers the device’s saturation output current. And, this contributes to enhancing the device’s short-circuit current-withstanding capability, as further analyzed in subsequent sections.

When two studied MOSFETs are compared in the reverse conduction mode, as shown in the third quadrant of Figure 4b, the body diode in the conventional MOSFET turns on at 2.7 V. While, in the proposed one, the MOS-channel diode starts to turn on at 1.2 V. A lower turn-on voltage lessens the possibility of bipolar degradation in SiC MOSFETs by suppressing the turn-on of the body diode as well as reducing conduction losses.

**Figure 5:**
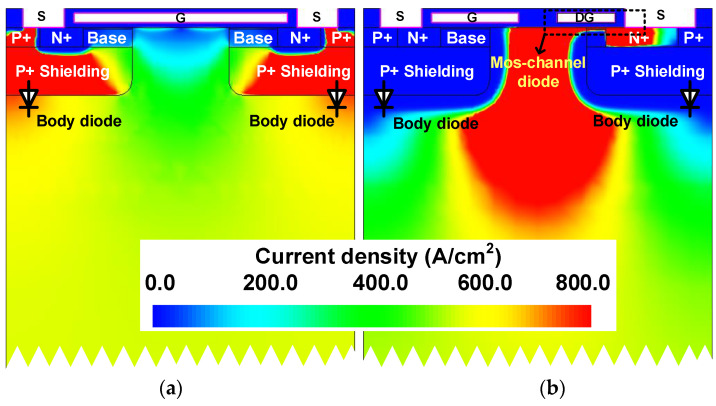
Total reverse current density distribution at *I*_SD_ = 11 A (550 A/cm^2^) of the (**a**) conventional MOSFET and (**b**) the proposed MOSFET.

**Figure 6:**
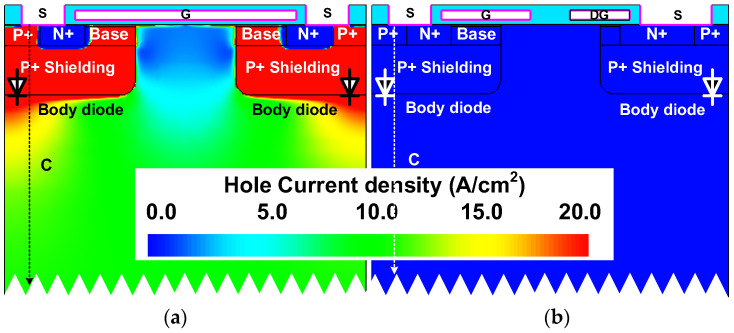
Hole current density distribution at *I*_SD_ = 11 A (550 A/cm^2^) of the (**a**) conventional MOSFET and (**b**) the proposed MOSFET.

**Figure 7:**
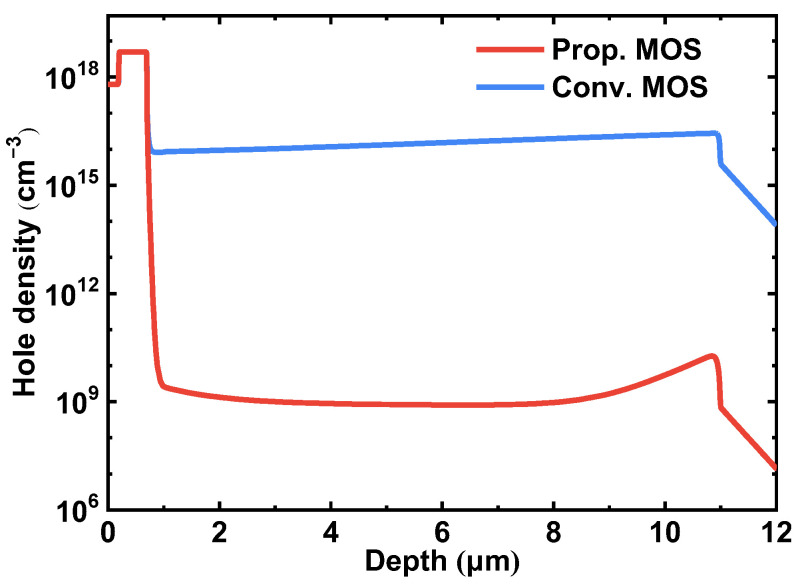
Distribution of the hole density along line C in Figure 6 of the two studied MOSFETs.

The source-to-drain current density distributions at a rated current of 11 A (550 A/cm^2^) of both studied devices are compared in Figure 5. Unlike the Conv. MOS, the body diode is inactivated, and the reverse current is conducted through the MOS-channel diode in the proposed MOSFET. Figure 6 shows the hole current density distributions of the two studied MOSFETs. The Prop. MOS can prohibit the hole injection from the P^+^ shielding region into the n-type layer. To further demonstrate this, the hole density along with line C are revealed in Figure 7. The hole density in the drift region of the proposed MOSFET is in the order of around 10^8^, while the conventional one is 10^16^. The extremely low minority carrier concentration in the drift region of the proposed device demonstrates the inactivation of hole–electron recombination, which will trigger bipolar degradation. 

**Figure 8:**
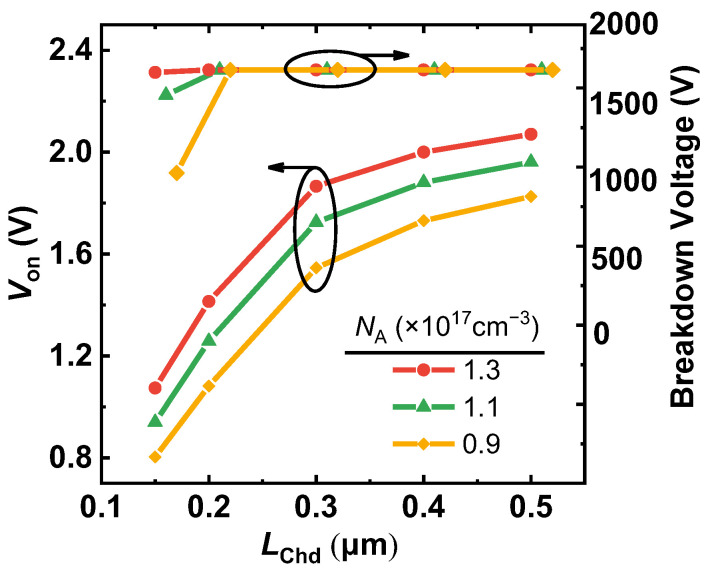
The effect of *L*_Chd_ on *V*_on_ and breakdown voltage of the proposed MOSFET with different *N*_A_.

The influence of *L*_Chd_ and the doping concentration of P-base (*N*_A_) on the cut-in voltage (*V*_on_) and the BV of the proposed MOSFET are shown in Figure 8. As in formula (2), the smaller *N*_A_ and shorter *L*_Chd_ will both reduce the *V*_on_ because of the lower electron potential barrier of the MOS-channel diode. As a result, when *L*_Chd_ is shortened from 0.5 μm to 0.2 μm, *V*_on_ is decreased from 2.0 V to 1.2 V. However, *L*_Chd_ should not be too small, because below 0.2 μm, the blocking capability of the device will degenerate. *V*_on_ increases gradually with *L*_Chd_ and *N*_A_. Even if *L*_Chd_ is increased to the Conv. MOS channel length, *V*_on_ is still decreased by 0.7 V, from 2.7 V to 2.0 V. This is due to the dummy gate’s ability to reduce the potential barrier. Furthermore, when *L*_Chd_ is greater than 0.2 μm, *L*_Chd_ and *N*_A_ will have no influence on BV.

**Figure 9:**
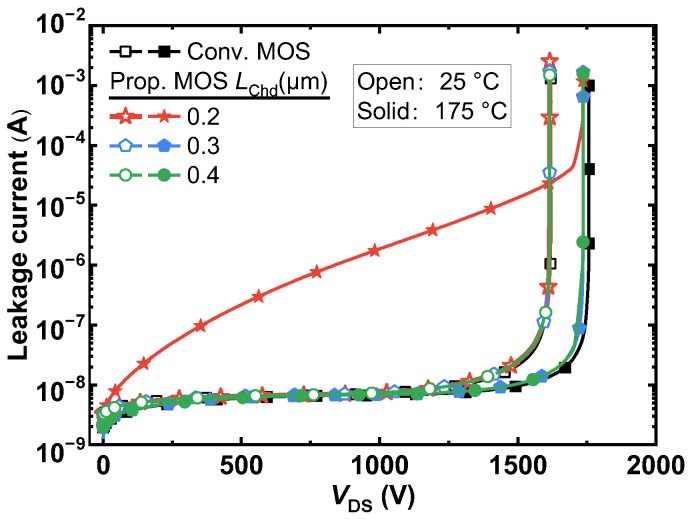
The blocking characteristics of the two studied SiC MOSFETs at room and elevated temperatures.

**Figure 10:**
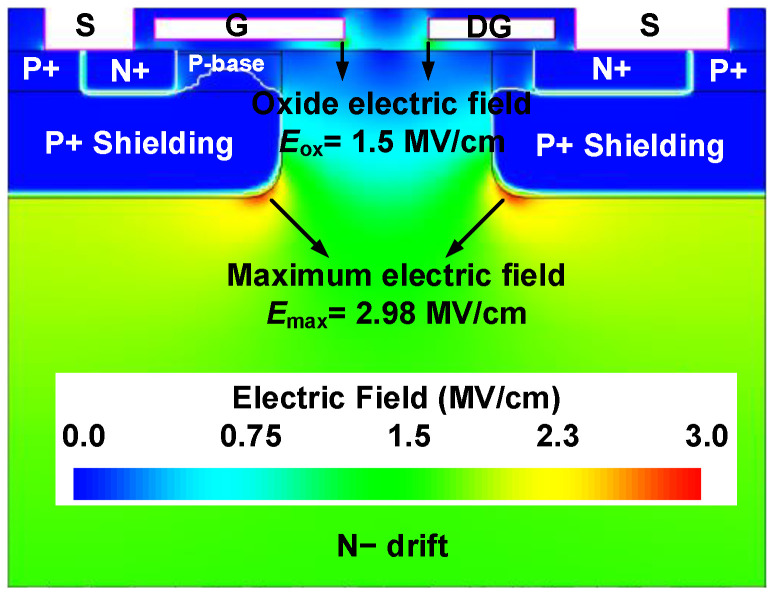
The off-state breakdown electric field distribution of the Prop. MOS.

Figure 9 shows the breakdown curves at room temperature and elevated temperature (up to 175 °C) of the conventional and proposed MOSFETs. It can be seen that the Prop. MOS has almost the same BV as the Conv. MOS because the high electric field is protected by the P^+^ shielding region and the MOS-channel diode has nearly no influence on the BV. In addition, they exhibit the same leakage current at room temperature. When *L*_Chd_ is 0.2 μm, however, the high-temperature leakage current begins to rise linearly as the reverse-biased voltage increases. This is because the electron potential barrier of the MOS-channel diode is extremely low. Therefore, it is necessary to give up some of *V*_on_’s benefits and choose structural parameters with an *L*_Chd_ bigger than 0.2 μm when the proposed MOSFETs must be used in a continuous high-temperature application. At room temperature, the electric field distribution of the proposed MOSFET at the breakdown voltage is shown in Figure 10. The highest electric field, *E*_max_, is located at the corner of the P^+^ shielding layer and measures 2.98 MV/cm. It can be observed from this figure that the device exhibits a uniform electric field distribution, with the maximum gate oxide field, *E*_ox_, located at the corners of the polysilicon gate and measuring 1.5 MV/cm. This value is below the safe limit of 3 MV/cm for maximum gate oxide field [2], indicating that the introduced MOS channel diode in the Prop. MOS does not significantly degrade the breakdown characteristics of the device.

**Figure 11:**
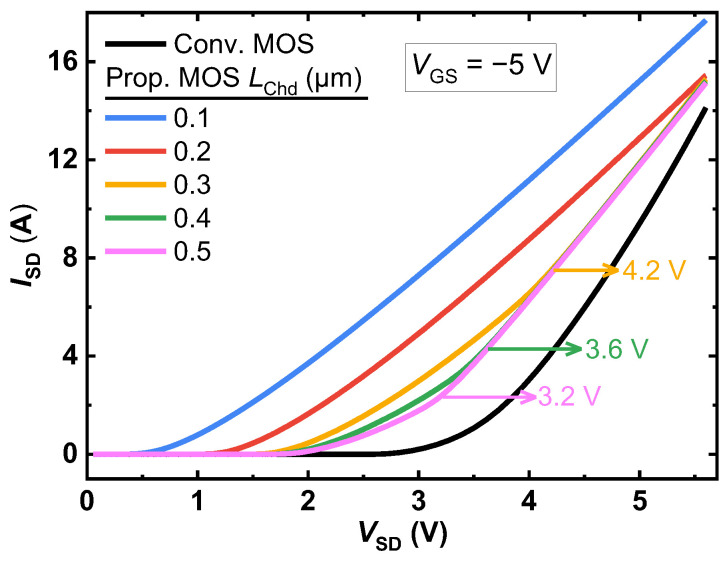
Comparison of the third-quadrant conduction characteristics of the two studied MOSFETs.

Figure 11 plots the third-quadrant conduction characteristics of the two studied MOSFETs. The parasitic body PiN diode of the Conv. MOS is open at 2.7 V. For the proposed MOSFET, a smaller *L*_Chd_ results in a smaller *V*_on_ and a smaller channel resistance of the MOS-channel diode. Accordingly, when *L*_Chd_ is decreased, the inflection in the *I-V* curves that indicates the bipolar conduction of the PiN body diode is also postponed. As a result, when *L*_Chd_ is reduced from 0.5 μm to 0.3 μm, the inflection point is improved by 1.0 V (from 3.2 V to 4.2 V). When *L*_Chd_ = 0.2 μm, the parasitic body PiN diode is suppressed until a very high current of 15.4 A (770 A/cm^2^) is reached and the corresponding inflection point is more than 5.6 V. This suggests that decreasing *L*_Chd_ can reduce the resistance and barrier height of the MOS channel diode, thereby reducing the overall resistance of the low barrier current path. Consequently, it raises the turn-on voltage of the PiN body diode, improving the device’s ability to resist bipolar degradation.

**Figure 12:**
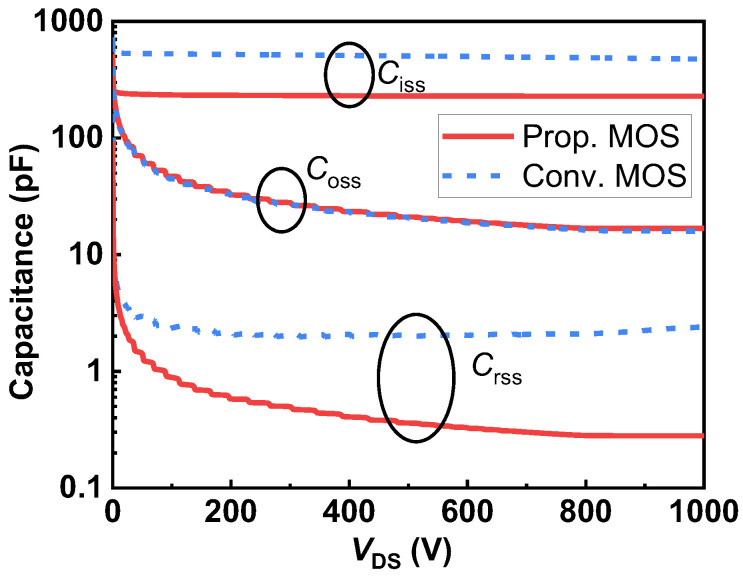
Comparison of proposed SiC MOSFET and conventional SiC MOSFET parasitic capacitance.

**Figure 13:**
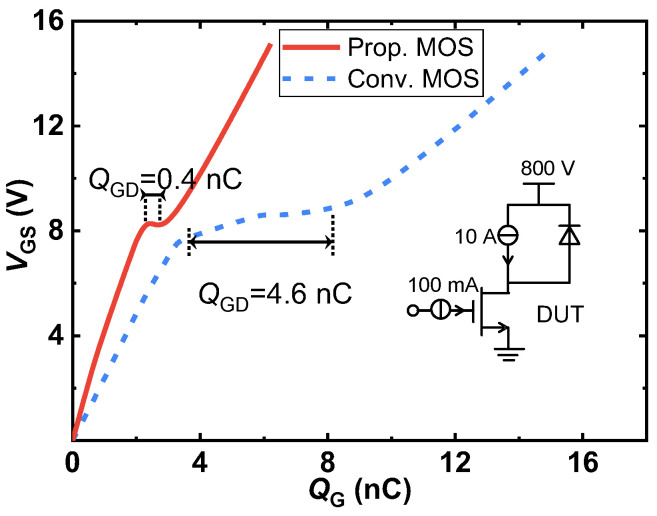
Gate charge characteristics of the proposed MOSFET and the conventional MOSFET; the test circuit configuration is shown in the inset.

It is well-known that the switching speed is constrained by the device capacitance. The input capacitance affects the pace of switching state transitions, whereas the gate-drain capacitance regulates the rate of change of drain current and voltage. Figure 12 shows the input, output, and reverse-transfer capacitances of the two studied SiC MOSFETs at a frequency of 1 MHz. The output capacitances (*C*_oss_) of the two MOSFETs under study are nearly equal, as shown by the comparison results, because the junction capacitance between the P^+^ shielding region and drift region is the same. However, the proposed device’s input capacitance (*C*_iss_) is found to be 53.2% lower than that of the conventional MOS at *V*_DS_ = 800 V because the overlapping area between the gate electrode and oxide is reduced. Importantly, the Prop. MOS possesses a much lower reverse-transfer capacitance (*C*_rss_ or *C*_GD_) compared with the conventional one. The *C*_rss_ of the Prop. MOS at *V*_DS_ = 800 V is dropped by 86.7% from 2.10 pF to 0.28 pF. This is due to the use of split-gate technology, which reduces the overlapping area between the gate electrode and the drift region, lowering gate-to-drain capacitance and charge [31]. The gate charge (*Q*_G_) and the gate-to-drain charge (*Q*_GD_) in the proposed MOSFET are also drastically reduced, as shown in Figure 13, and the test circuit can be seen in the inset. *Q*_G_ and *Q*_GD_ for the Prop. MOS are 6.2 nC and 0.4 nC, respectively, with a drain supply voltage of 800 V and a current of 10 A, and it achieves a 59.7% improvement in *Q*_G_ and a 91.3% improvement in *Q*_GD_ compared to the Conv. MOS. Accordingly, HF-FOM1 (*R*_on,sp_ × *Q*_GD_) and HF-FOM2 (*R*_on,sp_ × *C*_GD_) are improved by 8.8× and 5.7×, respectively.

**Figure 14:**
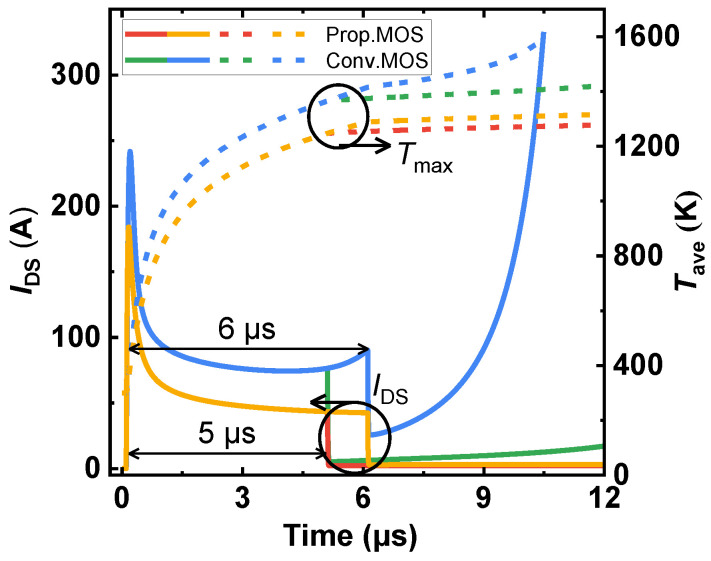
Simulated short-circuit waveforms of the two studied SiC MOSFETs. The short-circuit condition is *V*_GS_ of 15 V, *R*_G_ of 10 Ω, and *V*_DS_ of 800 V.

Figure 14 shows the short-circuit (SC) behaviors of two studied MOSFETs using electro-thermal simulations (*V*_GS_ of 15 V and *V*_DS_ of 800 V). The *I*_DS_ and average lattice temperature (*T*_ave_) waveforms indicate that the Prop. MOS has superior SC ruggedness [35,36]. This is because the MOS-channel diode lowers the effective channel density. The peak drain current of the Prop. MOS is thus 23.7% lower than that of the Conv. MOS (decreases from 241 A to 184 A), despite the increased *R*_on,sp_. As a result, the SC withstand time of the Prop. MOS is higher than the Conv. MOS, which failed for thermal runaway at 6 μs.

**Figure 15:**
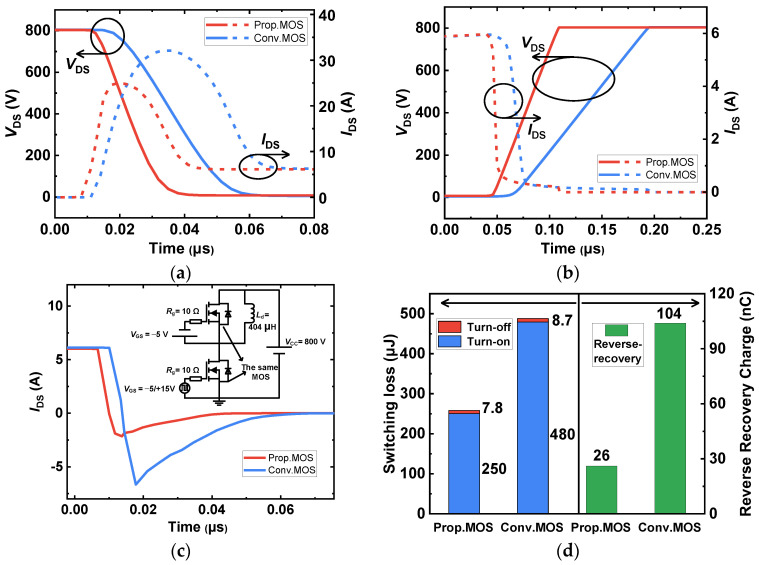
Switching characteristics of (**a**) turn-on waveforms, (**b**) turn-off waveforms, (**c**) reverse recovery waveforms of the body diodes and (**d**) calculated switching loss and diode reverse recovery charge.

The switching performances of the investigated SiC MOSFETs were evaluated using double-pulse tests (DPT), as displayed in Figure 15. The turn-on and turn-off waveforms for the devices are depicted in Figure 15a,b, respectively. Due to the lower *Q*_GD_, it is remarkable that the Prop. MOS achieves the shorter switching time, which means a lower switching loss. The reverse recovery characteristics are also shown, as can be seen in Figure 15c, and the DPT circuit is provided in the inset. When the Prop. MOS acts as a freewheeling diode, its peak reverse recovery current and time are greatly reduced. This is because the injection of minority carriers for the proposed MOS has been declined. The switching loss (*E*_SW_) and the reverse recovery charge (*Q*_rr_) of the two devices are summarized in Figure 15d. The total switching loss of the Conv. MOS is 488.7 μJ, while for the Prop. MOS it is 257.8 μJ (reduced by 47.2%). The *Q*_rr_ of the proposed device is 26 nC, which is 75% lower than the conventional SiC MOSFET (104 nC). For comparison, Table 2 summarizes the key electrical properties at a temperature of 25 °C for the Conv. MOS and the Prop. MOS. As a result, the planar integrated MOS channel diode SiC MOSFET that has been proposed can reduce switching losses, effectively suppress the bipolar degradation effects from the PiN body diode, increase the device’s figures-of-merits, and preserve the good short-circuit withstand capacity, as shown by Table 2 and the discussion above.

## 4. Conclusions

In this paper, a novel split-gate SiC MOSFET with an integrated MOS-channel diode structure is proposed and demonstrated using numerical simulations. The low electron potential MOS-channel diode provides a current path when the device is working in the third quadrant. Therefore, the suggested SiC MOSFET satisfactorily resolves the bipolar deterioration concerns in addition to having a lower diode *V*_on_ than the intrinsic body diode. To further explain the barrier-lowering effect, a concise analytical model of the potential barrier is suggested. With the use of split-gate technology, the switching characteristics of the device are also improved. As a result, the high-frequency figure-of-merits of *R*_on,sp_ × *C*_GD_ and *R*_on,sp_ × *Q*_GD_ are improved by 5.7× and 8.8×, respectively. Although the *R*_on,sp_ of the novel device is slightly degenerated, the short-circuit capability is increased. Therefore, the superior performance makes the proposed SiC MOSFET a competitive candidate for external-diode free high-frequency electronics applications.

## Figures and Tables

**Table 1:** Device parameters used in simulations.

Device Parameters	Unit	Conv. MOS	Prop. MOS
Gate poly-Si width	μm	1.9	0.9
Source poly-Si width	μm	-	0.5
Gate/Source gap width (*L*_GS_)	μm	-	0.4
P-base depth	μm	0.2	0.2
P-base doping concentration	cm^−3^	1.1 × 10^17^	1.1 × 10^17^
JFET region width (*W*_JFET_)	μm	1.0	1.0
JFET region thickness	μm	1.0	1.0
P^+^ shielding region depth	μm	0.5	0.5
P^+^ shielding doping concentration	cm^−3^	5.0 × 10^18^	5.0 × 10^18^
Cell pitch	μm	3.6	3.6

**Table 2:** Summary of electrical characteristics for Conv. MOS and Prop. MOS.

	Conditions	Prop. MOS	Conv. MOS	Unit
*V*_on_	*V*_GS_ = −5 V, *I*_SD_ = 0.1 A	1.2	2.7	V
BV	*I*_DS_ = 1 μA	1612	1618	V
*R*_on,sp_	*V*_GS_ = 15 V, *I*_DS_ = 6 A	13.3	10.2	mΩ·cm^2^
*C*_GD,sp_	*V*_DS_ = 800 V, *f* = 1 MHz	14	105	pF/cm^2^
*Q*_G,sp_	*V*_DS_ = 800 V, *I*_DS_ = 10 A	310	755	nC/cm^2^
*Q*_GD,sp_	*V*_DS_ = 800 V, *I*_DS_ = 10 A	20	230	nC/cm^2^
*Q*_rr,sp_	*V*_DS_ = 800 V, *I*_DS_ = 6 A	1.3	5.2	μC/cm^2^
*E*_SW_	*V*_DS_ = 800 V, *I*_DS_ = 6 A	12.9	24.4	mJ/cm^2^
*R*_on_ × *C*_GD_	-	186.2	1068.9	mΩ·pF
*R*_on_ × *Q*_GD_	-	266	2341.4	mΩ·nC

## Data Availability

Not applicable.
